# Etiopathogenesis of Insulin Autoimmunity

**DOI:** 10.1155/2012/457546

**Published:** 2012-02-22

**Authors:** Norio Kanatsuna, George K. Papadopoulos, Antonis K. Moustakas, Åke Lenmark

**Affiliations:** ^1^Department of Clinical Sciences, Skåne University Hospital (SUS), Lund University, CRC Ing 72 Building 91:10, 205 02 Malmö, Sweden; ^2^Laboratory of Biochemistry and Biophysics, Faculty of Agricultural Technology, Technological Educational Institute of Epirus, 47100 Arta, Greece; ^3^Department of Organic Farming, Technological Educational Institute of Ionian Islands, 27100 Argostoli, Greece

## Abstract

Autoimmunity against pancreatic islet beta cells is strongly associated with proinsulin, insulin, or both. The insulin autoreactivity is particularly pronounced in children with young age at onset of type 1 diabetes. Possible mechanisms for (pro)insulin autoimmunity may involve beta-cell destruction resulting in proinsulin peptide presentation on HLA-DR-DQ Class II molecules in pancreatic draining lymphnodes. Recent data on proinsulin peptide binding to type 1 diabetes-associated HLA-DQ2 and -DQ8 is reviewed and illustrated by molecular modeling. The importance of the cellular immune reaction involving cytotoxic CD8-positive T cells to kill beta cells through Class I MHC is discussed along with speculations of the possible role of B lymphocytes in presenting the proinsulin autoantigen over and over again through insulin-carrying insulin autoantibodies. In contrast to autoantibodies against other islet autoantigens such as GAD65, IA-2, and ZnT8 transporters, it has not been possible yet to standardize the insulin autoantibody test. As islet autoantibodies predict type 1 diabetes, it is imperative to clarify the mechanisms of insulin autoimmunity.

## 1. Introduction

**Figure 1:**
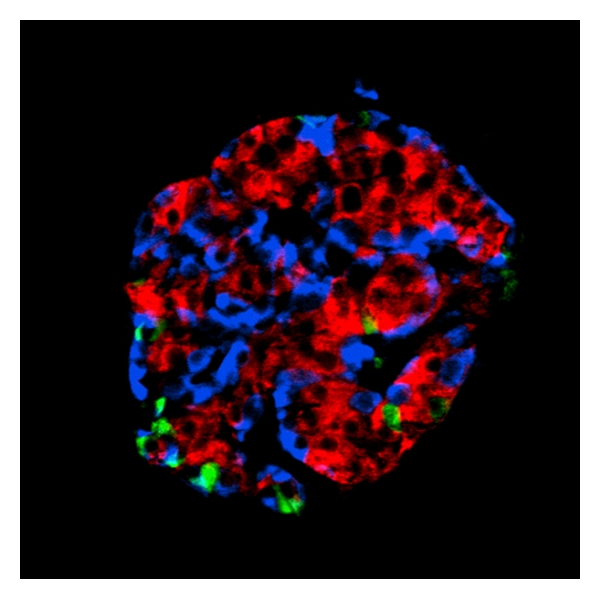
Immunocytochemistry of human islet cell subtypes. An isolated human islet is shown after immunocytochemical staining with antibodies against insulin (red), glucagon (blue), and somatostatin (green). Note that the architecture of human islet endocrine cells are distinctly different from that of rats and mice where glucagon cells occupy the mantel of the islets. Published with courtesy of Erik Renström.

**Figure 2:**
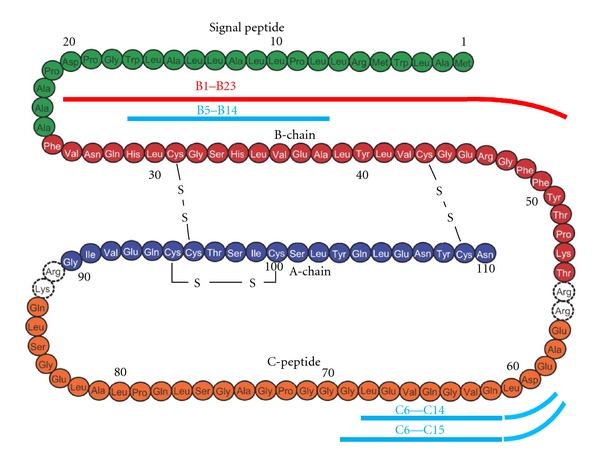
The primary structure of human preproinsulin. The B1–B23 peptide bound to HLA-DQ8 (A1*03:01-B1*03:02) is shown in red and can be found in Figure 3(a). The peptides bound to HLA-A2 as illustrated in (b), (c), and (d) are shown in blue for C6–C14, C6–C15 as well as for B5–B14.

The pancreatic islets constitute about 2-3% of the pancreas weight that is about 100 grams in adults [1]. The islets represent the endocrine portion of the pancreas and are present as more than a million well-defined cellular clusters throughout the pancreas [2, 3]. Each pancreatic islet (Figure 1) is composed of about 54% beta cells, 35% alpha cells, and 11% delta cells in addition to connective tissue and capillary cells [4]. Proinsulin, converted to insulin (Figure 2), is the major hormone produced in the beta cells while glucagon and GLP-1 are produced by the alpha cells, somatostatin by the delta cells, and pancreatic polypeptide by the PP cells. Pancreatic islet cells are also reported to produce ghrelin [5], apelin [6, 7], and CART [8–10]. These polypeptide hormones may be coexpressed with insulin in the beta cells or with other hormone-producing cells [8]. PP cells are more often seen in the head of the pancreas, while alpha cells dominate the tail [11, 12]. Insulin is the life-saving hormone for people suffering from type 1 and at times type 2 diabetes (see what follows). More beta cells are available than necessary to main blood glucose at normal levels. However, loss of insulin has catastrophic consequences. It has been estimated that 50% of the pancreas may be removed by surgery without a development of diabetes [13, 14]. Type 1 diabetes (T1D) is an autoimmune disease leading to a progressive loss of beta cells as they are attacked by the patients' own immune system (for reviews see [15–18]). T1D has a prodromal stage of islet autoimmunity. Children who develop islet autoantibodies against all four autoantigens: insulin, GAD65, IA-2, or ZnT8 (Table 1), before 3–5 years of age, tend to have a shorter prodrome prior to the clinical onset than older children, young adults, or adults [19]. These individuals may have multiple islet autoantibodies for years before the clinical onset of the disease [20]. GAD65, not insulin, autoantibodies characterize patients with latent autoimmune diabetes in adults (LADA) [15–18]. It has been estimated that although an individual may be positive for islet autoantibodies for months to years, the clinical onset does not occur until 80–90% of the beta cells have been killed [21]. Hence, T1D appears due to the selective autoimmune destruction of the pancreatic beta cells [16, 22]. The major genetic factor for T1D is the HLA-DQ locus on chromosome 6p21 [23]. Recent reviews can be found in [24, 25]. The association between the HLA Class II genes and T1D is well established and several HLA-DQ genotypes have been used to randomize newborn children to follow up investigations of the development of islet autoantibodies [26–30]. All over the world, the majority (80–90%) of newly diagnosed T1D children do not have a first-degree relative (father, mother, or sibling) already affected by the disease. The presence of certain HLA-DQ already at birth confers the genetic risk for T1D (Table 2). The highest risk is conferred by the HLA-DQ2/8 genotype. The risk for T1D with this genotype is highest in the young but is markedly decreasing with increasing age [31, 32]. Affected sib-pairs with T1D share HLA alleles more often than expected, and alleles at the Class II DR and DQ loci are not only associated with susceptibility to but also negatively associated with T1D and therefore offer at least partial protection [33]. In a large population-based study the HLA DQ A1*01:02-B1*06:02 (DQ6.2) was rarely found among T1D children below the age of 10; however, the negative association was decreased with increasing age and lost at 30 years of age [34]. It is noted that other HLA genotypes, often with somewhat similar physicochemical properties confer T1D risk in other populations such as in Japan and China (Table 2) [35–39]. As indicated the risk for T1D conferred by HLA-DQ is dependent on age. It is therefore important that autoantibodies against insulin are not only present particularly in young children at the time of clinical diagnosis of T1D but also prior to the clinical onset [17, 40, 41]. As will be reviewed the autoimmune reaction against insulin in T1D has been mapped in terms of both cellular [42, 43] and humoral [17, 44] recognition. However, insulin is a target not only in T1D but also in other autoimmune conditions. In Hirata's disease insulin autoantibodies are detected in association with hypoglycemia in the patient [45]. This disease is also associated with HLA Class II (Table 2) [46, 47]. The detailed mechanisms by which patients recognize their own insulin as an autoantigen may therefore have vastly different consequences for the patient and these differences will be discussed in the present paper. The reader is referred to the following reviews where insulin autoimmunity in T1D [17, 48, 49] or in the insulin autoimmune syndrome [47, 50, 51] has previously been reviewed.

**Note**

The first crystal structure of a human pathogenic TCR in complex with HLA-A2—InsS15-23 has been determined, and the TCR orientation with respect to the HLA-A2—peptide complex is diagonal [52, 53].

## 2. Insulin in the Etiology of Type 1 Diabetes

T1D may be viewed as a two-step disease. The first step is the initiation of islet autoimmunity; the second step is precipitation of diabetes when islet autoimmunity has caused a major *β*-cell loss (>80%), and insulin deficiency becomes clinically manifest. The pancreatic beta cells are destroyed in an aggressive autoimmune process. The immunopathogenesis of T1D is associated with T-lymphocyte autoimmunity, and the disease is often referred to as a T-cell-mediated disease [79–81]. This is somewhat self-evident as an immune response cannot be initiated without the help from CD4+ positive T-helper cells. Also it is rare that an immune response does not engage all cells in the immune system as cytotoxic CD8+ T cells are not able to develop without the help from CD4+ T-helper cells. These cells are also critical for the activation of B cells to differentiate into autoantibody-producing plasma cells. The importance of both T and B cells in the pathogenesis of T1D is illustrated in recent clinical trials [82, 83]. Monoclonal antibody therapeutics, depleting T cells (CD3 antibodies) or B cells (CD20 antibodies; Rituximab), had similar effects to transiently inhibit the progression of beta-cell loss after the clinical onset of T1D measured as residual beta-cell function [82–86].

Studies of children who have been followed from birth indicate that autoantibodies against insulin often appear before GAD65, IA-2, or ZnT8 autoantibodies [87, 88]. So far, it is not known whether CD4+ or CD8+ T cells specific for insulin can be detected in the peripheral blood prior to the appearance of insulin autoantibodies. Reactivity of CD4+ and CD8+ T cells towards insulin as well as preproinsulin (Figure 2) epitopes was reported both in newly diagnosed and in long-term patients [62, 69, 70, 89–91]. The reactivity of CD4+ T cells has been recorded mostly in connection with the susceptibility alleles HLA-DR3, HLA-DR4, or both, and only rarely in connection with HLA-DQ alleles [43, 65–67, 92–97]. A wide variety of reactivities to the preproinsulin (Figure 2) molecule have been reported. It is remarkable that even CD4+ T cells specific for posttranslationally A6Cys-A7Cys disulfide-linked insulin have been detected [65]. This also extends to the mouse reactivities where most H2-A/E alleles appear to have very strongly binding epitopes to the proinsulin molecule [98]. By contrast, the reactivity of CD8+ T cells is not linked to any particular HLA-A/B/C allele. A large variety Class I molecules bind insulin epitopes presented in T1D patients to sensitized T cells leading *in vivo* to proinflammatory cytokine secretion (IFN*γ*, TNF*α*) and cytotoxicity to beta cells [69, 70, 89–91]. It is remarkable that a protein of only 110 amino acids contains so many epitopes for a large spectrum of HLA Class I and Class II alleles. It should be noted that insulin autoantibodies may also react with proinsulin. Hence, it cannot be excluded that the triggering autoantigen is proinsulin rather than insulin. Still, it remains to be clarified why IAA is strongly associated with islet autoimmunity in the very young.

In laboratory mice attempts were made to answer this question. NOD mice unable to express the insulin 1 and insulin 2 genes were given a mutated proinsulin transgene in which tyrosine on residue 16 in the B chain was changed to alanine. This mutation abrogated the T-cell stimulation of insulin autoreactive T-cell clones. Female mice with only the altered proinsulin did not develop insulin autoantibodies, insulitis, or diabetes. It was suggested that the proinsulin/insulin molecules have a sequence that is a primary target of the insulin autoreactivity in the spontaneously diabetic NOD mouse. The conclusion was that the insulin peptide B(9–23) might be an essential target of the immune destruction of the NOD mouse [99]. It is important to note that the InsB9–23 sequence (identical in mouse I, mouse II, and human insulins) is a combitope, that is, a combination of epitopes: the H2-K^d^-specific InsB15–23 [100] and the I-A^ g7^-specific InsB12–20 (at the endosomal pH of 5.5 [101]). In the first case, B16 tyrosine is the anchor at pocket B of this very weakly binding peptide, and the alanine substitution leads to no binding and no recognition by the respective T-cell clone; hence, such cells are not even selected in the thymus or the periphery. In the second case, the tyrosine residue on position B16 would be the prime TCR contact residue p5, so its substitution with alanine would most likely result in no recognition at all. 

The observation in gene-manipulated laboratory mice may be relevant to human T1D as B(9–23)-specific T cells could be demonstrated in freshly isolated lymphocytes from patients with recent-onset T1D as well as from subjects at high risk for the disease [62]. In humans the register for binding of the InsB9–23 peptide to the four major HLA-DQ alleles conferring susceptibility to T1D is identical and represented by InsB13–21, which is only slightly different from the InsB12–20 in NOD mice [63, 101]. It was speculated that these insulin autoreactive cells may contribute to the T1D disease process as the T cells produced the proinflammatory cytokine IFN-*γ* [62]. This observation led to a clinical trial with an altered peptide ligand of the InsB9–23 epitope, where B16Tyr and B18Cys were changed to Ala [102]. The trial was unsuccessful and one of the reasons may be found in subsequent studies of another autoimmune disease, multiple sclerosis: the orientation of the cognate T-cell receptor from pathogenic myelin basic protein-specific and HLA-DRB1*15:01-, HLA-DRB5*01:01-, or -DQB1*05:02/A1*01:02-restricted CD4+ T cells was strikingly different from the canonical diagonal one seen in the case of recognition of complexes of microbial peptides with MHC II molecules [103–105]. Essentially, in all three cases the TCR was tilted to recognize elements of the MHC II molecule and the N-terminus of the bound peptide (mostly up to position 5); also, TCR recognition was sensitive to the alanine-substitution of a very limited set of residues and surprisingly tolerant of many nonconservative substitutions at these positions, indicating perhaps remarkable flexibility by the self-reactive TCR in adjusting to the substitutions in order to maintain productive binding to the complex. Any future trials with altered peptide ligands must take these facts into account [106].

Current investigations in humans at risk for T1D still do not answer the question what factor may trigger the insulin autoimmunity. Numerous studies during more than 100 years suggest that virus infection of beta cells may explain the induction of islet autoimmunity. It has been suggested based on experiments in laboratory mice that virus infection and replication in beta cells will eventually lyses these cells. Following the lytic event, virus as well as beta-cell debris will be engulfed by local dendritic cells or by macrophages. Cells that physiologically die of apoptosis (e.g., during development) express on the outer leaflet of their cell membrane phosphatidyl serine that is specifically recognized by dendritic cells (the most effective of APC) and apoptotic cells are engulfed in a way leading to tolerance. In case of an infection, however, the pattern recognition receptors (known as Toll-like receptors, TLR) present in all nucleated cells, are activated upon contact with specific microbial components; hence, the engulfment of virally infected cells under aberrant conditions may not lead to immunological tolerance [107–109].

APC engulfing virally infected pancreatic islet beta cells will be activated as they process the cell debris. The activated APC will move through the lymphatics to the draining lymph nodes of the pancreas. Hence, presentation of beta cell autoantigen (as well as of the virus antigens) will take place in the lymph nodes rather than in the pancreatic islets themselves. In the lymph nodes the APC will present islet autoantigen including insulin to the T-cell receptor (TCR) of CD4+ T-helper cells. Insulin is by far the most plentiful protein in the beta cell, as a single human beta cell contains about 12 pg of this protein. Once such a CD4+ T-helper cell with an insulin-specific TCR is activated, it will induce an immunological reaction against insulin by recruiting both CD8+ cytotoxic T cells and also antibody-producing B cells. Pancreatic tissue from six T1D donors revealed that Coxsackie B4 enterovirus could be demonstrated in the islets in three of the six diabetic patients. The infection was indeed specific to beta cells. However, the data indicted a nondestructive islet inflammation mediated mainly by natural killer cells [110]. It is possible therefore that the destruction of virus-infected beta cells is mediated initially by the innate immune system. The NK-cell-mediated destruction may in turn stimulate the regulated immune system to develop islet autoimmunity but only in subjects with certain HLA-DQ genotypes (Table 2).

An alternative hypothesis is that dietary cow's milk insulin could trigger beta-cell autoimmunity [111]. A primary immune reaction against bovine insulin would be the trigger of an immune reaction towards human insulin. However, when analyzing enterovirus infections in relation to the consumption of cow's milk formula, there seemed to be an interaction between these two factors in inducing islet autoimmunity [112].

## 3. Type 1 Diabetes with Islet Autoantibodies

Most patients with T1D have islet autoantibodies at the time of clinical diagnosis. Autoimmune diabetes rather than T1D would therefore be a more appropriate designation of the disorder. Several authors have reported that about 10–15% of newly diagnosed patients with diabetes classified with T1D have no islet autoantibodies at the time of clinical onset. The question will then arise if patients have had islet autoantibodies, which disappeared prior to the clinical onset. The use of autoantibody tests against ICA, insulin, GAD65, IA-2, and the three variants of ZnT8 as well as islet cell antibodies (ICAs) by indirect immunofluorescence, in more 600 newly diagnosed T1D children, indicates that only 5% did not have any of the seven different types of autoantibodies [113]. It was not possible to determine if these children have had autoantibodies and lost them prior to diagnosis. However, in children born to mothers with islet autoantibodies during pregnancy, it was found that such children tended to be negative at the time of clinical onset [114]. Presence of islet autoantibodies at birth may explain why some T1D children are islet autoantibody negative at clinical diagnosis [114].

In Japan, a distinct subtype of T1D characterized by a rapid clinical onset (duration of symptoms before presentation and insulin treatment may be days and usually no longer than two weeks) and without islet autoantibodies has been established as fulminant type 1 diabetes mellitus (FT1DM) [115]. FT1DM, recently reviewed in [116, 117], is considered to have the following three diagnostic criteria: (1) rapid occurrence (within 7 days) of diabetic ketosis or ketoacidosis after the onset of hyperglycemia symptoms (polydipsia, polyuria and fatigue). Often patients have elevated ketone bodies in the urine and serum at presentation; (2) plasma glucose levels would be ≥16.0 mmol/L but glycated hemoglobin level < 8.5% at presentation; (3) urinary C-peptide excretion <10 *μ*g/day or fasting serum C-peptide level < 0.3 ng/mL (<0.10 nmol/L) and <0.5 ng/mL (<0.17 nmol/L) after intravenous glucagon (or after meal) tested within 1-2 weeks after presentation. Ancillary criteria include the absence of autoantibodies against islet autoantigens such as insulin, GAD65, and IA-2 [116, 118]. Serum pancreatic enzyme levels (amylase, lipase, or elastase-1) were found to be elevated in 98% of the patients. Flu-like symptoms (fever, upper respiratory symptoms, etc.) or gastrointestinal symptoms (upper abdominal pain, nausea, vomiting, or both, etc.) precede disease presentation in 70% of the patients [119]. The disease may also occur during pregnancy or just after delivery [116, 120]. As a part of the Japanese nationwide survey of FT1DM, it was found that the Class II HLA-DR4-DQ4 (DRB1*04:05-DQB1*04:01) haplotype was significantly more frequent in patients with FT1DM [121]. Interestingly enough, the HLA immunogenetics of pregnancy-associated FT1DM may differ. In a recent study it was reported that the haplotype frequency of HLA-DRB1*09:01-DQB1*03:03 was significantly higher in pregnancy-associated FT1DM compared to both FT1DM not associated with pregnancy as well as to controls [122]. Both biopsy and postmortem immunocytochemical investigations at onset revealed an infiltration of T lymphocytes and macrophages [123] in and around pancreatic islets. Mononuclear cell infiltrations were also found in the exocrine portion of the pancreas [123, 124]. A detailed postmortem histopathological investigation of three patients revealed that macrophages and T cells but no natural killer cells had infiltrated the islets and the exocrine pancreas [125]. In addition, enterovirus may be present in beta cells in association with several markers of innate system activation and cytokine expression [126, 127]. It was speculated that a strong inflammatory reaction may explain the rapid loss of beta cells. Although islet autoantibodies were negative at the time of clinical diagnosis, GAD65 autoantibodies appeared transiently in some patients following the initiation of insulin treatment [128]. To our knowledge there are no studies investigating the possible appearance of insulin autoantibodies or insulin antibodies (developing after insulin treatment). FT1DM was also described in patients in Korea [129], China [130], and France [131]. Further studies of possible FT1DM patients in other countries are needed.

## 4. Antigen Presentation of Insulin by HLA-DQ

Insulin autoantibodies are primarily detected in children below the age of 5 years [15–17]. In Kappa statistics of agreement there was a moderate to fair agreement between any pairs of autoantibodies against GAD65, IA-2, or ZnT8 (W,R,Q) (Table 1), while insulin autoantibodies showed only a slight agreement with any combination [113]. It is often observed that insulin autoantibodies are the first to appear, at least in children younger than 3–5 years of age. However, it has been difficult to dissect the sequence of events that leads to the formation of insulin autoantibodies in very young children. One could envisage the following scenario. Beta cells would be killed, perhaps lysed by a virus infection. The dead beta cells or remnants thereof would be engulfed by APC. These cells are activated and migrate through the lymphatic system to the lymph nodes that drain the pancreas. Antigen presentation to CD4+ T cells would take place in the lymph node. It is possible that the antigen presentation is particularly effective in small children leading to an early insulin autoantibody response [34]. This hypothetical mechanism is consistent with studies in experimental animals [132, 133].

The APCs are expected to process preproinsulin (associated with remnants of the endoplasmic reticulum), proinsulin, or insulin to peptides, which may be picked up by HLA-DR, DQ, or both, heterodimers in the small lysosomal-like transition vesicles. The higher affinity proinsulin/insulin peptides will replace the invariant chain peptide (CLIP) that “protects” the groove, and the resulting trimolecular complex is eventually presented on the APC surface. The appearance of insulin autoantibodies was found to be associated with HLA-DQ8 as well as with the regulatory region of the insulin gene (INS VNTR) on chromosome 11 [34]. Molecular studies have aimed at identifying which insulin peptides might possibly be presented by HLA-DQ and -DR heterodimers on APC [134, 135]. In fact, insulin peptides from the A chain have been identified as high affinity binders to HLA-DRB1*04:03, an allele associated with protection from autoimmune diabetes in the high-risk HLA-DQ2/8 heterozygotes [64]. T cells oligoclonally expanded from pancreatic draining lymph nodes obtained from long-term T1D patients recognized the insulin A1-15 epitope and were restricted by DR4 [42]. Yet these clones required high amounts of insulin peptides to proliferate, so it is not clear what stage of the pathogenesis they represent.

**Figure 3:**
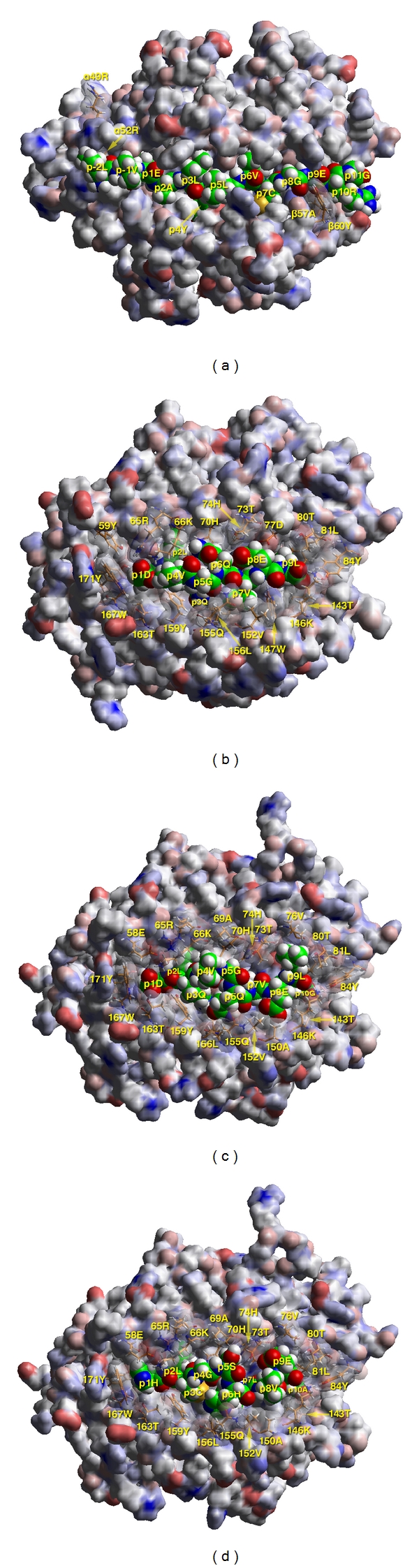
(a) Insulin B1–B23 epitope modeled into the *α*1*β*1 domain of the HLA-DQ8 heterodimer. It has been shown that CD4+ T cells from type 1 diabetes patients are sensitized to this complex [62]. The insulin peptide is in space-filling form with its atoms colored as follows: carbon, green; oxygen, red; nitrogen, blue; hydrogen, white; sulfur, yellow. The HLA-DQ8 (A1*03:01-B1*03:02) heterodimer is in van der Waals surface representation, colored according to atom charge (red, negative; blue, positive; gray, neutral; partial charges in shades in-between). A few residues from the HLA-DQ molecule in contact with the antigenic peptide are shown via a transparency function in stick form (same color notation as in the peptide with the exception of carbon that is in orange). Modeling and binding studies have shown that the insulin peptide binds to the other three HLA-DQ diabetes-susceptible haplotypes (A1*05:01-B1*02:01, A1*05:01-B1*03:02, A1*03:01-B1*02:01) in an identical register [63]. This view is as seen from the T-cell receptor, which might fit with its symmetry axis in an approximate diagonal fashion with respect to the peptide axis (fitting of TCRs specific for microbial peptides). The few examples of structures of autoimmune TCR in complex with cognate MHC II-peptide complexes reveal an off-diagonal recognition involving mostly the N-terminal half of the peptide and more selective contacts with the MHC II molecule. Molecule drawn from coordinates provided in [136]. (b) TCR view of the complex of HLA-A2, the most frequent Class I allele among Caucasians, with the proinsulin peptide C6–C14 (D**L**QVGQVE**L**; anchors in bold). Color code and conventions are as in (a). The peptide shown is part of epitope pool 60 (AED**L**QVGQVE**L, **ED**L**QVGQVE**L, **D**L**QVGQVE**L**, and **L**QVGQVE**L**). All four epitopes should bind well to HLA-A2, with SI < 3 in controls and SI > 3 in 3/6 T1D patients [91]. The epitope depicted was first identified, though not tested on PBMCs of T1D patients in [68]. (c) TCR view of the complex of HLA-A2, with the insulin peptide C6–C15 (D**L**QVGQVEL**G**, anchors in bold). Color code and conventions are as in (a). This is part of epitope pool 61 (ED**L**QVGQVEL**G, **D**L**QVGQVEL**G, L**QVGQVEL**G**, and Q**V**GQVEL**G**). The first three of the epitopes should bind weakly to HLA-A2 and the fourth one hardly at all. It is also possible to have a different register altogether, especially for the last two peptides** (**LQ**V**GQVEL**G **and Q**V**GQVEL**G**), with SI < 2 in all controls and SI > 3 in 2/6 T1D patients [106]. (d) TCR view of the complex of HLA-A2 with the insulin peptide B5–B14 (H**L**CGSHLVE**A**), recently identified as an epitope for HLA-A2 in type 1 diabetes patients of recent onset, with the very sensitive tetramer labeling using the quantum dot technique [71]. This peptide belongs to pool 30 [106] (QH**L**CGSHLVE**A, **H**L**CGSHLVE**A, L**CGSHLVE**A**), where it has also shown reactivity. Note that this peptide will also bind very strongly to the protective allele HLA-DQB1*06:02, as well as to the slightly susceptible allele HLA-DQB1*06:04, in the same core nonamer register B6–B14 [139, 140].

As previously noted, the insulin B13–B21 nonamer core binds in the same register to all of the four HLA-DQ haplotypes in the HLA-DQ2/8 heterozygote: A1*05:01-B1*02:01, A1*03:01-B1*03:02, A1*03:01-B1*02:01, and A1*05:01-B1*03:02 [63, 136–138]. It is not possible to speculate whether these four particular trimolecular complex epitopes would allow degenerate recognition by a single TCR on CD4+ T cells (Figure 3(a)). One could easily note, however, that as far as reactivity to insulin is concerned, the dominant protection conferred by HLA-DQB1*06:02 concerns its high affinity binding in the register of InsB6-14 [63]. The high affinity binding would result in denying (stealing) this epitope from any of the lower affinity binding diabetes-susceptible alleles. Such high affinity binding may induce regulatory T cells that could prevent the initiation of autoimmunity by diabetogenic T cells [63]. It is important to note that the recognition of the trimolecular complex (DQA1 chain—insulin peptide—DQB1 chain) by CD4+ T cell TCRs is likely to represent the very initiation of an autoimmune response to (pro)insulin as an autoantigen. The presentation of the insulin peptide in a DQ8 trimolecular complex would represent the very first initiation of an immune response to insulin. The first responder cells are expected to be CD4+ T-helper cells [137]. Such cells have already been found in the peripheral blood of newly diagnosed patients reactive with the InsB9–23 peptide [62]. Insulin-specific CD4+ T-helper cells would in turn help both CD8+ T-cytotoxic cells as well as B cells expressing autoantibodies recognizing insulin. CD8+ T-cytotoxic cells would be expected to express a TCR recognizing an insulin peptide presented on HLA Class I molecules on the beta-cell surface (Figures 3(b)–3(d)).

## 5. Antigen Presentation of Insulin by Class I HLA-A, B, or C

CD8+ T-cytotoxic cells directed against insulin peptides expressed on MHC Class I have been described both in the NOD mouse and in man [100, 101, 141, 142]. Remarkably, the two NOD mouse epitopes InsB15-23 and InsB25-C34, respectively, bind either very weakly or very strongly to the restriction element, H2-K^d^ [70, 142]. The preproinsulin epitopes to several HLA-A/B alleles (including the most frequent allele in the Caucasian population, -A2) (Figures 3(b)–3(d)) span the entire molecule, and the frequency of reactivity to the different epitopes varies [69, 70, 89–91]. Remarkably, occasional high responses to certain peptides are also seen in controls (SI > 4), with no other sign of autoimmunity [91]. In a pioneering study on *in situ* reactivity of persons at onset of type 1 diabetes and patients with long-standing disease, it has been shown that CTLs in HLA-A2^+^ individuals showed reactivity to single epitopes from 6 different autoantigens (preproinsulin included, epitope 15–23). There was an inversely proportional staining of pancreases with HLA-A2 tetramers with respect to age from diagnosis. In fact, no such reactivity was detected in any patients with over 10 years time from the date of onset of type 1 diabetes [143].

## 6. Antigen Presentation of Insulin by B Lymphocytes

**Figure 4:**
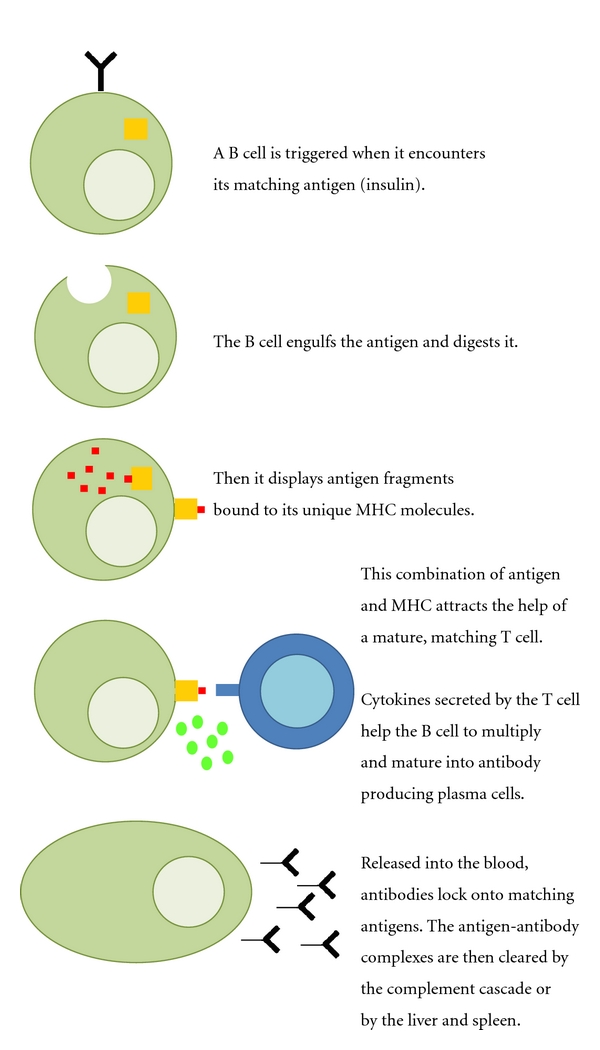
Schematic representation of possible mechanisms by which insulin (or proinsulin) may be processed by B lymphocytes. The B lymphocyte as viewed as a professional antigen-presenting cell (APC).

An alternative pathway to the formation of IAA is illustrated in Figure 4. This pathway remains to be fully explored in humans. The clinical trial with Rituximab (CD20 monoclonal antibody) in newly diagnosed T1D children demonstrates that depletion of B lymphocytes was associated with a significant preservation of mixed meal-stimulated C-peptide [83]. The contribution of B lymphocytes to T1D pathogenesis may have been overlooked. As illustrated in Figure 4, B lymphocytes with an antigen receptor recognizing insulin would take up the insulin and process it to be presented on HLA-DR, -DQ, or both. The trimolecular complex with insulin would next be recognized by a TCR on the surface of a mature, matching CD4+ T-helper cell. Upon the cell-to-cell contact the CD4+ T-helper cell is activated to produce cytokines (such as IL-4 or IL-10). These cytokines would help the B cell to differentiate, replicate, and mature into an IAA producing B lymphocyte, and eventually turning into a plasma cell. It is important to note that Rituximab treatment appeared to reduce antibody formation to new antigens such as the bacteriophage PhiX174 [144]. It was suggested that Rituximab decreased both antibody production and isotype switching [144]. However, at the same time as residual C-peptide was preserved [83], Rituximab suppressed IA but not the levels of postdiagnosis GADA, IA-2A, and ZnT8A [145]. In the European-Canadian cyclosporine trial, it was demonstrated that cyclosporine reduced the formation of insulin antibodies in response to the regular insulin therapy given to all the participating T1D patients [146]. It is of interest in this regard that Rituximab-treated patients were thought to be able to develop immunological tolerance to bacteriophage PhiX174 [144]. In Stiff Person Syndrome, Rituximab was reducing GADA in some [147] but not in all [148] patients. Further studies are warranted to determine the interaction between APC, T-helper cells, and B lymphocytes. It needs to be established to what extent B lymphocytes may be acting as APC that either initiate, maintain, or both, the autoimmune response to insulin in children.

## 7. Analysis of IAA and Standardization of IAA Assays

Measurement of IAA was initially limited by the large serum volume required for the early immunoprecipitation assays, which used polyethylene glycol to separate immune complexes [149]. The first IAA assay required one milliliter serum or plasma [150]. Insulin was labeled by ^125^I in an approach similar to that which had been used for both regular insulin radioimmunoassays as well as for insulin-receptor-binding experiments [151]. Later it was found that labeling of multiple tyrosine residues compromised both antibody—as well as receptor binding [152]. These observations resulted in the now established use of only insulin that is monoiodinated at position A14 [153]. The improvement in insulin iodination procedures [154, 155] made it possible to develop alternative radiobinding assays that required less serum. This type of microassay allowed a major reduction of the amount of serum used and has improved assay specificity [156, 157].

The development of alternative assays continues especially as the international standardization workshops demonstrate significant interlaboratory variability [158, 159]. In the first workshops it was found that IAA could not be detected in an ELISA type of assay [160]. The IDW [161] and DASP [158, 159] international workshops to standardize IAA continued to demonstrate that there was a poor interlaboratory consistency in the IAA assay [149, 156, 157, 162]. IAA determination varies more between laboratories compared to other diabetes autoantibodies such GADA and IA-2A. The Diabetes Antibody Standardization Program (DASP) has improved and standardized measurement of IAA associated with T1D [158].

## 8. IAA before the Clinical Onset of Diabetes

The IAA radioimmunoassay was first tested in serum or plasma samples from siblings to first-degree relatives with T1D. These siblings, including monozygotic twins or triplets, were followed longitudinally for the appearance of IAA and other islet autoantibodies [163, 164], in larger prospective studies such as BABYDIAB [87, 165], DIPP [29, 166], and DAISY [167, 168]. IAA was reported to show an association between levels and risk for T1D, which was not observed for GADA or IA-2A [168]. These observations seemed also to be corroborated in studies of children at genetic risk for T1D based on HLA typing rather than having a first-degree relative with the disease [169]. In the Diabetes Prevention Trial-1 (DPT-1) [170], GADA, IA-2A were measured along with ICA and IAA [170]. No subjects with IAA as single autoantibodies developed T1D [170]. When a second autoantibody appeared, any other autoantibody except IAA was added significantly to the prediction of T1D [170]. In the DIPP study of children born with high-risk HLA, IAA tended to be the first autoantibody to appear [166]. It is therefore possible that the initiation of the T1D disease process may involve insulin itself or proinsulin, perhaps also preproinsulin [20, 41, 87, 171]. However, most authors suggest that the number of islet autoantibodies is the strongest predictor of clinical onset of T1D [170]. It can however not be excluded that IAA affinity may be a better predictor for T1D in children with multiple autoantibodies [74, 172]. Indeed, high-affinity cell surface antibody on B lymphocytes readily promotes their differentiation and proliferation upon antigen binding, in contrast to low-affinity antibody [173].

## 9. Are IAA Epitopes Related to Proinsulin Peptides Presented on HLA Class I or II Heterodimers?

There is a paucity of detailed investigations to clarify IAA epitopes of proinsulin and insulin (Table 3). There is a lack of information to what extent HLA-DQ or DR are associated with IAA binding to either A chain, B chain, or proinsulin autoantibody epitopes. Similar to HLA Class I peptide binding (Figures 3(b)–3(d)), IAA was reported to recognize the A8–A10 (13) epitope [74, 76, 77]. It is not clear why IAA would recognize the same epitope as might be presented on HLA Class I molecules. The B1–B3 [78] as well as the B3 position [72, 73], both presented on HLA Class II molecules (Table 2) may also be recognized by IAA. Studies with systematic site-directed mutagenesis of the preproinsulin cDNA may prove useful to map the IAA binding site more carefully in relation to the HLA-DQ and DR genotypes of newly diagnosed, non-insulin-treated T1D patients, or IAA-positive nondiabetic subjects. Such studies are also important as it has been suggested that the IAA levels may be the best predictor of clinical onset in young children [168] as well as in children born to mothers with T1D in the BABY DIAB study [87]. Further studies are also needed to determine epitope specificity in relation to the apparent polyclonal nature of IAA and their similarity to the insulin antibodies (IA) detected after insulin therapy has been initiated [174, 175].

## 10. Insulin Antibodies after the Diagnosis of Type 1 Diabetes

The diagnostic sensitivity of IAA for T1D is on the average only about 30% [34] but varies with the age at onset. In children below the age of 3 years the diagnostic sensitivity may be as high as 50–60% [113] but decreases to about 10% in T1D patients diagnosed after 20 years of age. It was estimated that IAs produced in response to the insulin treatment appear after about 7 days [34]. When comparing binding characteristics between IAA and IA, it was found that the two antibody types were comparable in several affinity tests [174]. The authors therefore concluded that both IAA and IA were polyclonal in nature and that both developed in response to insulin as the antigen [174]. In some individuals, it is therefore possible that insulin itself is able to break the immunological tolerance to allow the formation of IAA. It follows that it cannot be excluded that insulin treatment itself may induce a T1D disease process. For example, insulin given to type 2 diabetes patients in Japan was thought to induce T1D [176]. In these patients insulin antibodies (IAs) of high titer were detected at or after the development of insulin deficiency. These IAs were characterized by an extremely high-affinity and a very low-binding capacity. The characteristics of these insulin-treatment-induced IA were thought to be similar to the IA found in the insulin autoimmune syndrome [176]. The insulin aspart had comparable immunogenicity to human insulin, and antibodies developing in response to either insulin seemed to cross-react [177–180]. High titer insulin antibodies requiring immunosuppression have been reported [181]. Epitope-specific insulin antibodies may develop in some patients who showed benefit when one insulin analogue was replaced by another [181–183]. Similar to animal insulins, also insulin analogues such as Lispro insulin may cause insulin allergy [184].

## 11. The Insulin Gene in Type 1 Diabetes and Possible Mechanisms of Tolerance Induction

The insulin gene (INS) region is an established T1D susceptibility locus. The variable nucleotide tandem repeat (VNTR) in the promoter region of the insulin gene may contribute to T1D possibly by mechanisms of central tolerance [185]. The INS VNTR is composed of 14 to 15 bp variant repeats. The shortest (Class I) variable number of tandem repeat (VNTR) alleles was found to increase, whereas the longest (Class III) alleles were observed to decrease in the patients in comparison to the controls [185]. The possible role of central tolerance is illustrated by the observation that IAAs in newly diagnosed T1D patients was found to be associated with the INS VNTR polymorphism in some [34, 186] but not all studies [187]. In children born to mothers with T1D, it was reported that the combination of genotyping for high-risk HLA-DQ (e.g., HLA-DQ2/8 and 8/8) and INS VNTR identified a minority of children with an increased T1D risk [188]. One study compared the INS VNTR polymorphism between Finland and Sweden [189]. The T1D risk genotypes (Class I/I and I/III) were significantly more common in Finland than in Sweden, both among patients and controls. Class III homozygous genotypes showed varying degrees of protective effect due to polymorphisms within Class III. These observations suggest that heterogeneity between protective Class III lineages could exist.

However, it is important to note that the frequency of disease-associated Class I haplotype is markedly high (>90%) in the Japanese general population [190]. While comparisons of risk may be evaluated in high incidence countries such as Finland and Sweden, this will be difficult in the Japanese population. However, a meta-analysis suggested that the Class I haplotype as such was significantly associated with T1D in Japanese [190].

It was reported that most Class III alleles are associated with higher levels of INS transcription than Class I alleles in the thymus [191, 192]. Higher levels of INS expression in the thymus may promote negative selection of insulin-specific T lymphocytes, which may play a critical role in the pathogenesis of T1D [193]. Studies of the human thymus is complicated by the relative inaccessibility of this tissue as further studies are needed to establish a possible relation between INS VNTR genotypes, thymic expression of preproinsulin, and a negative selection of (prepro)insulin-specific T lymphocytes. It is interesting to note that in one of the few studies on the subject thus far the INS VNTR Class III allele, in a homozygous or heterozygous state, has been shown to promote regulatory type IL-10-producing CD4+ T-cell responses [194].

Recent research has indicated that tolerance to insulin and other tissue-specific proteins is affected via a number of different mechanisms. For one, such proteins are continuously produced and presented via MHC I and II proteins in the thymus as well as in peripheral lymphoid organs (spleen and lymph nodes) [195–197]. One of the important transcription factors promoting the expression of many such proteins (including insulin) is AIRE (autoimmune regulator) [198]. In fact, a mutation in AIRE leads to autoimmune polyendocrine syndrome-(APS-) 1 with symptoms of parathyroidism, Addison's disease, and candidiasis, and invariably to autoantibodies to interferon-*ω* [199]. AIRE is expressed both in the thymus and in peripheral organs and controls to variable extents the expression of several tissue-specific proteins in the thymus and in the lymph nodes. It seems that in the latter organ in mice, both endothelial cells as well as fibroblastic reticular cells express in subsets a number of different such proteins (e.g., tyrosinase, GAD67, and retinal S-antigen) [197, 200]. The impaired expression of alpha-myosin in the thymus in mice and humans was shown to be intimately linked to autoimmunity in the heart [201]. It is essential that all T1D autoantigens, especially insulin, be tested in humans, for expression both in the thymus and in the lymph nodes, in order to better understand the possible mechanisms of immune tolerance. The second important advance in the field of immune tolerance has been the “rediscovery” of the former suppressor T cells, now called regulatory T cells, first of CD4+ lineage, but subsequently of CD8+ lineage as well [202, 203]. These cells are characterized by contact-obligatory inhibition of proliferation of T-effector cells, and their development and maintenance is dependent on TGF*β* and the transcription factor FoxP3. In fact, mutations in the FoxP3 gene (located on the X-chromosome) lead to the IPEX (immune dysfunction, polyendocrinopathy, enteropathy, X-liked) syndrome, characterized by stillbirth or food allergy, diarrhea, and several endocrine autoimmune disorders, with neonatal T1D as most prominent [204, 205]. Remarkably, in one such case with IPEX syndrome and neonatal diabetes, bone marrow transplant at 18 months of age resolved the immune deficiency and reduced the daily insulin requirement [206]. T-regulatory cells have received a lot of attention in connection with T1D. It has been shown that patients with the disease (newly diagnosed or of long standing) have Tregs with insufficient regulatory capacities, while there has been a disagreement regarding their absolute deficiency, owing mostly to the different ways of defining the phenotype of these cells [207–209]. The inability of Tregs from T1D patients to regulate the activity of diabetogenic T cells has been attributed to the nonsusceptibility of the latter cells to regulation [210, 211]. It will be interesting to see if in any way the induction and maintenance of insulin-specific Tregs can alter the antigen-specific Th1-prone cytokine response to a regulatory type, avoiding thus the outcome of T1D [212]. Remarkably, CD8+ T-regulatory cells specific for preventing GAD65 autoreactivity at the T-cell level have already been shown to exist in control subjects and be deficient in diabetic patients [213]. In a retrospective study of pancreatic tissue from patients who died soon after clinical presentation of T1D, it was noted that no FoxP3+ Tregs could be detected, while other immunocytes were plentiful in insulin-bearing islets [214], a finding whose significance has yet to be assessed.

## 12. Insulin Autoimmune Syndrome

The insulin autoimmune syndrome (IAS, Hirata disease) is characterized by a combination of fasting and sometimes postprandial hypoglycemia, high serum concentrations of total immunoreactive insulin, and presence in the serum of polyclonal autoantibodies against native human insulin [45, 215].

It is noted that IAS has a strong genetic predisposition and the majority of the IAS patients were reported from Japan, where it is the third leading cause of hypoglycemia. Predisposition to IAS is strongly associated with DRB1*04:06 [46, 47]. It is also known that some drugs with sulphydryl groups in their chemical structures can induce the formation of insulin autoantibodies in predisposed individuals [51]. A patient with Graves' disease who has the haplotype HLA-Bw62/Cw4/DR4 with a specificity for DRB1*04:06 may be at risk of developing IAS after administration of methimazole [216]. Since 2003, a rapidly increasing number of patients with alpha-lipoic-acid-induced IAS have been reported [51, 217–219]. Generally in Japan, alpha-lipoic acid has gained popularity as a supplement for dieting and antiaging since 2004 [51]. Although critical amino acid residue(s) for insulin antigen presentation on the DRB1*04:06 molecules have been identified [46], the mechanisms that trigger this presentation and the subsequent chronic autoimmune response that generates both polyclonal and monoclonal insulin autoantibodies remain to be clarified.

## 13. Summary and Future Directions

Humoral autoimmunity against insulin, first described in 1983 is established but needs to be better defined. The current IAA radioimmunoassay continues to perform poorly in the DASP [158, 159]. The current interlaboratory variation is simply too large to allow valuable comparisons between laboratories throughout the world. The autoreactivity against (pro)insulin also needs to be better defined. It will be necessary to clarify the way by which (prepro)insulin released from dead or damaged beta cells is taken up by APC, processed and finally presented on HLA-DR and -DQ molecules. Is antigen-presentation by—DQ more critical than—DR to induce autoreactivity? Once IAAs have been formed, it will be critical to define the role of IAA-producing B cells and plasma cells. What is the role of B cells as APC in the disease process? Both CD4+ T-helper and regulatory T cells specific for (pro)insulin need also to be identified in humans at risk for T1D; the former have been shown to exist at the population but not at the clonal level. Insulin-specific CD8+ T cells may be critical to identify in children at increased risk for T1D as such children tend to develop IAA early, while CD8+ Tregs may be able to control the action of diabetogenic self-reactive immunocytes. It will be important to compare the insulin autoantibodies IAS with the IAA in T1D. The IAS is characterized by a combination of fasting hypoglycemia, high concentration of total serum immunoreactive insulin, and presence of autoantibodies to native human insulin in serum [46]. The release of insulin from the IAS insulin autoantibodies may cause hypoglycemia, and further studies are needed to explain why this type of autoantibodies may be related to hypoglycemia with no apparent loss of beta cells.

The view of insulitis has been revised: it is no longer considered an initiating phenomenon but rather the end stage of prolonged subclinical presence of islet autoantibodies including IAA. To what extent do insulin-specific CD8+ T cells contribute to insulitis? The possible effect of insulin treatment as a trigger or accelerator of autoimmune (type 1) diabetes needs further exploration. Why is it that insulin treatment may induce insulin dependency in Japanese type 2 diabetes patients, but not in others? Efforts need to be made to answer the nagging question whether insulin administration accelerates the loss of beta cells in Caucasian T1D patients.

## Figures and Tables

**Table 1:** Islet autoantigens in type 1 diabetes.

Autoantigen	Autoantibody	Present before clinical onset	
Insulin	Insulin autoantibodies	IAA	+
Proinsulin	Proinsulin autoantibodies	PAA	?
Glutamic acid decarboxylase, 65 kD		GAD65A	+
Insulinoma antigen-2		IA-2A	+
ZnT8 transporter		ZnT8A	+

**Table 2:** HLA haplotypes in the general population conferring significant risk for islet autoimmunity and type 1 diabetes.

HLA-DQ	References
(a) Type 1 diabetes	

*Europeans*	
A1*03:01-B1*03:02	[54, 55]
A1*05:01-B1*02:01	[56]
*French native*	
DRB1*04:05-DQA1*03:03-B1*03:02	[57]
*Mediterranean*	
DRB1*04:05-DQ A1*03-B1*02	
*Black populations*	
DRB1*07-DQ A1*03-B1*02	
*Japanese (haplotypes)*	
DRB1*04:05-DQA1*03:01/02?-DQB1*04:01	[35–37]
DRB1*09:01-DQA1*03:02-DQB1*03:03	[35–37]
DRB1*08:02-DQA1*03:01-DQB1*03:02	[36, 37]
DRB1*04:05-DQA1*03:01-DQB1*03:02	[37]
DRB1*03:01-DQA1*05:01-DQB1*02:01	[37]
DRB1*11:01-DQB1*03:01	[36]
DRB1*11:01-DQB1*03:02	[36]
DRB1*13:02-DQB1*06:04	[36]
*Koreans (haplotypes)*	
DRB1*03:01-DQB1*02:01	[58]
DRB1*04:01-DQB1*03:02	[58]
DRB1*04:05-DQB1*03:02	[58]
DRB1*04:07-DQB1*03:02	[58]
DRB1*04:05-DQB1*04:01	[35]
DRB1*09:01-DQB1*03:03	[35, 58]
*Chinese (haplotypes)*	
DRB1*09:01-DQA1*03:01/02-B1*03:03	[38]
DQA1*03-DQB1*03:03	[39]
DQA1*03-DQB1*04:01	[39]
DQA1*05-DQB1*02:01	[39]
*Indians (haplotypes)*	
DRB1*03:01-DQA1*05:01-B1*02:01	[59]
DRB1*04:01/02/04/05-DQA1*03:01/02-B1*02:01	[38]
*Sub-Saharan Africa (haplotypes)*	
DRB1*03:01-DQA*05:01	[60]
DRB1*04-DQA*03	[60]
DRB1*04-DQB*03:02	[60]
DRB1*03:01-DQB*02:01	[60]
DQA*05:01-DQB*02:01	[60]
DQA*03-DQB*03:02	[60]
*Latin America (haplotypes)*	
DRB1*04:05-DQB1*03:02	[61]
DRB1*03:01-DQB1*05:01	[61]
DRB1*04:01-DQB1*03:02	[61]
DRB1*04-DQA1*03:01-DQB1*03:02	[61]

(b) Insulin autoimmune syndrome (haplotypes)	

DRB1*04:06-DQA1*03:01-B1*03:02	[46, 47]

**Table 3:** Proinsulin and insulin peptides recognized by HLA-DQ, HLA-DR, and HLA-A2 compared to the epitopes recognized by IAA.

Binding molecule	Proinsulin/insulin sequence	Reference
HLA—Class II		
DQ8 (B1*03:02)	B9–B23	[62]
DQ8c (A1*03:01-B1*03:02)	B13–B21	[63]
DQ2c (A1*05:01-B1*02:01)	B13–B21	[63]
DQ2t (A1*03:01-B1*02:01)	B13–B21	[63]
DQ8t (A1*05:01-B1*03:02)	B13–B21	[63]
DQ6.2 (A1*01:02-B1*06:02)	B6–B14	[63]
DRB1*04:01	A1–A15	[42]
DR 04:01	C19-A3, C35-A19	[64]
DR 04:03	C35-A19, A8–A21	[64]
DR4	A1–A13	[65]
DRB1*04-DQ8	B11-C24, C28-A21, B20-C4, C18-A1	[66]
DRB1*04-DQ8	B1–B16, B11–B27, C13–C29	[67]

HLA—Class I		
A2	C6(5)–C14	[68]
	C6–C15	
A2 (A*02:01)	B10–B18	[69]
A2-B8	B10–B18	[70]
A1-B8	B17–B26	[70]
A2	B5–B14	[71]
A3	C22–C30, C25–C34	[71]
B7	S4–S13	[71]
A2	S15–23	[52]

IAA		
DRB1*04:06	B3 (asp)	[72, 73]
DRB1*04-DQB1*03:02	A8–A13	[74]
Unknown	Insulin, not proinsulin	[75]
Unknown	A8–A10	[76, 77]
Unknown	B1–B3, A17	
Unknown	B1–B3, A8–A13	[78]

c is cis and t is trans in transcription. S is signal peptide.

## Abbreviations

AIRE: Autoimmune regulator
APC: Antigen-presenting cells
APS: Autoimmune polyendocrine syndrome
CART: Cocaine and amphetamine regulated transcript
CD: Cluster of differentiation
CLIP: Class-II-associated invariant chain peptide
DAISY: Diabetes association in support of youth
DASP: Diabetes antibody standardization program
DIPP: Diabetes prediction and prevention
ELISA: Enzyme-linked immunosorbent assay
FoxP3: Forkhead box P3
FT1DM: Fulminant type 1 diabetes mellitus
GAD: Glutamic acid decarboxylase
GLP-1: Glucagon-like peptide-1
HLA: Human leukocyte antigen
IA: Insulin antibodies
IAA: Insulin autoantibodies
IAS: Insulin autoimmune syndrome
IA-2: Insulinoma-associated antigen-2
ICA: Islet cell antibody
IDW: International diabetes workshop
IFN: Interferon
IL: Interleukin
IPEX syndrome: Immune dysregulation, polyendocrinopathy, enteropathy, X-linked syndrome
MHC: Major histocompatibility complex
NK: Natural killer
NOD: Nonobese diabetic
TCR: T-cell receptor
TGF: Transforming growth factor
Th: T helper
TLR: Toll-like receptor
TNF: Tumor necrosis factor
Tregs: T-regulatory cells
T1D: Type 1 diabetes
VNTR: Variable number of tandem repeats
ZnT8: Zinc transporter isotype 8.
